# p75NTR Modulation by LM11A-31 Counteracts Oxidative Stress and Cholesterol Dysmetabolism in a Rotenone-Induced Cell Model of Parkinson’s Disease

**DOI:** 10.1007/s11064-025-04569-7

**Published:** 2025-10-04

**Authors:** Daniele Pensabene, Noemi Martella, Giuseppe Scavo, Emanuele Bisesto, Francesca Cavicchia, Mayra Colardo, Michela Varone, Sandra Moreno, Marco Segatto

**Affiliations:** 1 Department of Biosciences and Territory, University of Molise, Contrada Fonte Lappone, 86090 Pesche, Italy; 2 Department of Science, University Roma Tre, Viale Guglielmo Marconi 446, 00146 Rome, Italy; 3 Lab of Neurodevelopmental Biology, Neurogenetics and Molecular Neurobiology, IRCCS Fondazione Santa Lucia, Via del Fosso di Fiorano 64, 00143 Rome, Italy

**Keywords:** Cholesterol metabolism, Mitochondria, Neurodegeneration, Oxidative stress, Parkinson’s disease, P75NTR

## Abstract

The p75 neurotrophin receptor (p75NTR) plays a dual role in regulating both pro-survival and pro-apoptotic cascades in various physiological and pathological conditions, including within dopaminergic neuronal population. Notably, its overexpression has been documented in post-mortem Parkinson’s disease (PD) brains, where it correlates with a significant downregulation in neuroprotective intracellular mediators. In this study, we aimed at investigating the neuroprotective effects of p75NTR modulation by the small molecule LM11A-31 in a rotenone-induced neuronal model of PD. Differentiated SH-SY5Y cells were treated with 100 nM rotenone, with or without 500 nM LM11A-31. Our results show that LM11A-31 effectively mitigates PD phenotype by enhancing cell viability, reducing apoptosis, mitigating α-synuclein aggregation, and partially restoring neuromorphological features. Mitochondrial integrity was preserved, likely through the upregulation of transcription factors involved in mitochondrial biogenesis, namely PGC-1α and PPARs. LM11A-31 treatment also reduced oxidative damage to macromolecules, normalizing Nrf2 expression and enhancing protein S-glutathionylation. The antioxidant effect of p75NTR modulation may be partially attributed to the suppression of the NADPH oxidase regulatory subunits p22PHOX and p47PHOX. Additionally, LM11A-31 restored cholesterol homeostasis disrupted by rotenone, as evidenced by the increased NPC1 expression and lysosomal localization, normalized HMGCR levels, and reduced intracellular cholesterol accumulation. Collectively, these findings demonstrate that p75NTR modulation via LM11A-31 exerts neuroprotective effects by targeting key pathological features of PD, including oxidative damage, mitochondrial derangements, and cholesterol dysmetabolism, supporting its potential as a promising therapeutic tool in PD treatment.

**Supplementary Information:**

The online version contains supplementary material available at 10.1007/s11064-025-04569-7.

## Introduction

p75NTR was the first member of the tumor necrosis factor (TNF) receptor family to be identified [1]. This receptor is activated by the non-selective binding of neurotrophins (NTs), a small group of growth factors regulating development and function of the nervous system, by balancing neuronal survival and cell death [2, 3]. Both precursor and mature neurotrophins exert biological functions by interacting with p75NTR. Specifically, mature NTs bind p75NTR with low affinity to promote cellular survival and neurite outgrowth, whereas precursors preferentially bind to p75NTR to trigger apoptotic signaling cascades under physiological conditions [4, 5]. For this reason, p75NTR has emerged as a pivotal mediator of either survival or cell death, with its functional outcome depending on the nature of its ligands and co-receptor interactions. Altered expression or aberrant processing of p75NTR may result in massive cell death, contributing to the onset and progression of neurodegenerative diseases such as Alzheimer’s disease (AD) [6], Huntington’s disease (HD) [7] and Amyotrophic Lateral Sclerosis (ALS) [8].

Additionally, several studies demonstrated that p75NTR signaling governs dopaminergic cell survival and death under physiological conditions, suggesting a critical involvement of this receptor in the pathogenesis of Parkinson’s disease (PD) [9, 10]. This the most common neurodegenerative movement disorder, primarily caused by the progressive loss of dopaminergic neurons in the *Substantia Nigra pars compacta* (SNpc), leading to bradykinesia, resting tremor, and impaired motor control [11, 12]. While PD etiology remains quite elusive, common neurodegeneration hallmarks, such as cytoplasmatic inclusions of α-Synuclein (α-syn), mitochondrial dysfunction, oxidative stress, neuroinflammation and autophagy derangements, have been recognized [13, 14]. Emerging evidence points also to cholesterol dysmetabolism as relevant feature of PD physiopathology. Notably, a recent study has reported downregulation of Niemann-Pick C1 (NPC1) protein and the subsequent build-up of unesterified cholesterol within lysosomes in an in vitro model of PD, suggesting the concurrence of a Niemann-Pick type C disease (NPCD)-like phenotype and reinforcing the relevance of cholesterol alterations in PD [15].

A resolutive therapy for PD is not available. Currently, dopamine precursor levodopa (L-DOPA) remains the gold standard for symptomatic relief, as it increases dopamine concentration in the *striatum*. However, L-DOPA treatment is associated with both tolerance over time and drug-related adverse effects [16]. It is therefore imperative to seek new strategies to protect dopaminergic neurons against degeneration. In this context, p75NTR may represent a valuable target, as its activation has been shown to selectively favor dopaminergic neuronal death and downregulate neuroprotective transcription factors, thereby contributing to the progression of the neurodegeneration [17]. Accordingly, p75NTR overexpression correlates with the severity of dopaminergic cell death in a rat model of PD [11]. Additionally, p75NTR upregulation has been observed in a rotenone-based PD model, where it drives α-syn aggregation, possibly by enhancing its ubiquitination [18].

Emerging evidence also points to a role for the p75NTR signaling pathway in the regulation of redox homeostasis. Modulation of this receptor confers protection against oxidative injury induced by H₂O₂ and 6-OHDA, primarily through glutathione metabolism [19–21]. Furthermore, p75NTR has been proposed to function as a circadian regulator, promoting Nrf2 activation and, consequently, the transcription of antioxidant enzymes in a time-dependent manner [22].

p75NTR activation has also been implicated in the control of cholesterol metabolism. Specifically, its expression or stimulation is associated with the upregulation of genes involved in cholesterol biosynthesis [23, 24]. More recently, p75NTR activation has been reported to upregulate LDL receptor expression in both neuronal and hepatic cells, potentially via p38- and caspase-3–dependent mechanisms, thereby enhancing lipoprotein uptake [1, 25, 26]. Since both oxidative balance and cholesterol homeostasis are perturbed in PD, these findings further support the possible involvement of p75NTR in PD physiopathology.

Given the double-edged sword nature of p75NTR in regulating both cell death and survival, small molecules selectively modulating its activity have been developed for therapeutic purpose. These p75NTR-binding molecules, including THX-B, LM11A-24, and LM11A-31, efficiently suppress the activation of transduction pathways involved in cell death while concurrently enhancing trophic signaling. Particularly, LM11A-31 is specifically designed to bind p75NTR within the NGF loop1 binding domain and is the best characterized modulator to date. It is neither a strict agonist nor an antagonist, rather it facilitates pro-survival signaling induced by phosphoinositide 3-kinase (PI3K)/protein kinase B (PKB) while concurrently suppressing degenerative pathways [27].

The pharmacological modulation of p75NTR by LM11A-31 yielded promising results in AD and HD animal models and clinical trials, as it enhances neuronal resilience and slows the progression of pathophysiological features [7, 28, 29]. Despite these encouraging findings, the role of p75NTR pharmacological modulation in PD remains poorly understood.

Hence, the aim of this study was to evaluate the prospective beneficial effects of LM11A-31 in an in vitro model of PD by exposing differentiated SH-SY5Y cells to rotenone, a well-known environmental toxin that mimics the pathological phenotype. Particularly, this PD model was chosen since rotenone exposure faithfully reproduces key mechanisms of PD pathogenesis, such as build-up and aggregation of α-syn, progressive oxidative damage, apoptotic cell death and mitophagy impairment [30].

## Materials and Methods

### Cell Cultures

SH-SY5Y human neuroblastoma cell line was cultured at 5% CO_2_ in DMEM medium at high glucose (D6429, Merck Life Science, Milan, Italy), containing 10% (*v/v*) fetal bovine serum (FBS, F7524, Merck Life Science, Milan, Italy) and 1% penicillin/streptomycin solution (P06-07100, PAN Biotech, Aidenbach, Germany). Throughout the experiments, 250,000 cells were seeded; after 5 h, SHSY5Y differentiation was induced by incubating cells with DMEM, 1% FBS and 10 µM of retinoic acid (R2625, Merk Life Science, Milan, Italy) for 72 h. Then, cells were pre-treated either with p75NTR modulator LM11A-31 (100 nM or 500 nM, SML0664 Sigma-Aldrich) or with vehicle (DMSO, 1:1000 dilution in DMEM, FBS 1%). A concentration of 100 nM LM11A-31 was used as the reference dose, as it is frequently used and typically elicits a maximal response in similar in vitro studies [31–33]. After 24 h, cells were exposed to rotenone (100 nM, R8875, Merck Life Science, Milan, Italy) for other 24 h. We used a rotenone concentration of 100 nM, as this dosage falls within the range commonly employed to induce a Parkinsonian phenotype in various cellular models of PD [34–36]. To better assess mitophagy, cells were also treated with chloroquine (CQ, 20 µM, C6628, Merk Life Science, Milan, Italy) for 4 h to block the autophagic flux.

### Morphological Evaluation and Cell Count

SH-SY5Y cells were cultured in six-well plates and treated as abovementioned. Cells were observed and photographed employing Nikon Eclipse 7S10 optical microscope at a 20x magnification. Neurite length (expressed as arbitrary units), neurite network (sum of neurite lengths in each field) and neurite-bearing cells (expressed as ratio between the number of neurite-bearing cells and total cell number in the field) were analyzed with ImageJ software v.154d for Windows 10 (National Institutes of Health, Bethesda, MD, USA). Afterwards, cells were exposed to trypsin for 3 min and detached from the well surface. Samples were collected and cell count was conducted in Fast-Read 102^®^ (BVS100, Bio Sigma) chambers employing Nikon Eclipse 7S10 microscope. Three counts for each experimental group were performed.

### Cell Lysate and Western Blot Assays

SH-SY5Y were sonicated in sample buffer (Hepes 10 mM, KCl 10mM, MgCl2 1.5 mM, NP-40 0,1%, DTT 0,5mM, protease and phosphatase inhibitor cocktail) for 30 s to obtain a total cell lysate. Protein concentration was assessed with Lowry’s method and the Laemmli buffer was added for protein denaturation. The samples were boiled at 95 °C for 3 min. Twenty micrograms of protein extract were run employing SDS-PAGE electrophoresis. The Trans-Blot Turbo system (Biorad Laboratories, Milan, Italy) was used for protein transfer on nitrocellulose membranes. The membranes were later incubated at room temperature for 1 h with 5% fat free milk in DPBS (D1408, Merck Life Science, Milan, Italy, pH 7.4) and subsequently incubated with the primary antibody overnight at 4°C (Table 1). After incubation, membranes were washed with DPBS to discard excessive primary antibody and exposed for 1 h to HRP-conjugated secondary antibodies. Clarity ECL Western blotting (1705061, Bio-Rad Laboratories, Milan, Italy) was used for protein-antibody complex observation. Images were captured and densitometric analysis was carried out with ImageJ software v.154d for Windows 10 (National Institutes of Health, Bethesda, MD, USA). All samples were normalized for protein loading with GAPDH as housekeeping proteins. The suitability of GAPDH as a stable reference for protein loading normalization was confirmed through densitometric analysis, which revealed no statistically significant alterations in GAPDH levels among the experimental groups considered in this study. Densitometric calculations were expressed in arbitrary units, determined by the ratio between the protein band intensity and the respective housekeeping protein and normalized to the control levels. The antibody employed in this work for Western blotting are listed in Table 1.

**Table 1:** List of antibodies employed in this work

Antibody	Ref	Provider	Application
4-HNE	MA527570	ThermoFisher Scientific	IF 1:100
8-OH(d)G	sc-66,036	SantaCruz Biotechnology	IF 1:100
Catalase	sc-271,803	SantaCruz Biotechnology	WB 1:500
GAPDH	sc-32233sc-32,233	SantaCruz Biotechnology	WB 1:5000
GSH	Ab19534	Abcam	IF 1:150
Gpx 1	Ab22604	Abcam	WB 1:500
GSR	sc-133,245	SantaCruz Biotechnology	WB 1:500
GSS	sc-166,882	SantaCruz Biotechnology	WB 1:500
HMGCR	NBP2-61616	Novus	IF 1:300
LC3	L7543	Merck Life Science	IF 1:400
LRP1	sc-25,469	SantaCruz Biotechnology	IF 1:100
NOX2	sc-130,543	SantaCruz Biotechnology	WB 1:1000
NOX4	sc-518,092	SantaCruz Biotechnology	IF 1:50
NPC1	NB400-14855	NOVUS Biologicals	WB 1:2000
NRF2	sc-365,949	SantaCruz Biotechnology	IF 1:100
N-Tyrosine	sc-32,757	SantaCruz Biotechnology	IF 1:100
p22^phox^	sc-130,551	SantaCruz Biotechnology	IF 1:50
p47^phox^	sc-17,844	SantaCruz Biotechnology	IF 1:50
p75NTR	sc-271,708	SantaCruz Biotechnology	IF 1:100
Parkin	sc-32,282	SantaCruz Biotechnology	WB 1:500
PGC-1α	sc-13,067	SantaCruz Biotechnology	IF 1:50
Pink 1	sc-517,353	SantaCruz Biotechnology	WB 1:100
PPAR-α	Ab8934	Abcam	IF 1:300
PPAR-γ	sc-7273	SantaCruz Biotechnology	IF 1:100
SOD 1	MA515520	ThermoFisher Scientific	WB 1:500
SOD 2	sc-137,254	SantaCruz Biotechnology	WB 1:250
SQE	sc-271,661	SantaCruz Biotechnology	WB 1:500
SQS	sc-271,602	SantaCruz Biotechnology	WB 1:500
SREBP2	sc-13,552	SantaCruz Biotechnology	IF 1:40
TrxR1	sc- 28,321	SantaCruz Biotechnology	WB 1:300
α-synuclein	sc-12,767	SantaCruz Biotechnology	IF 1:50
γ-GCL	sc-390,811	SantaCruz Biotechnology	WB 1:500

### In Vitro Enzymatic Assays

After treatments, differentiated SH-SY5Y cells were collected and pellets were employed to perform Catalase and SOD activity using commercial kits (MAK531 and MAK528, Merck Life Science, Milan, Italy respectively) according to the manufacturer’s instructions.

### Immunofluorescence

Cells were seeded on sterilized and poly-L-lysine (P6282, Merck Life Science, Milan, Italy)-coated coverslips. After treatment, cells were fixed at room temperature either with 4% paraformaldehyde (D1408, Merck Life Science, Milan, Italy) solution in DPBS or with methanol (32215, Merck Life Science, Milan, Italy) for 5–10 min. Subsequently, cells were permeabilized for 5 min with DPBS containing Triton 0,1% (X100, Merck Life Science, Milan, Italy). In order to prevent non-specific antibody bounds, cells were later exposed to a 3% bovine serum albumin (BSA, A3912, Merck Life Science, Milan, Italy) solution in DPBS containing 0,1% Triton at room temperature for 1 h. Afterwards, cells were exposed overnight to primary antibodies (Table 1). The next day, samples were incubated for 1 h with either anti-mouse or anti-rabbit fluorescent conjugate antibodies (Table 1). DAPI (D9542, Merck Life Science, Milan, Italy) was used for nuclei staining. Coverslips were later mounted with Fluoroshield mounting medium (F6182, Merck Life Science, Milan, Italy) and visualized through confocal microscopy (TCS SP8, Leica Microsystems, Buccinasco, Italy) at 40× magnification. Images were acquired with LAS X software (version 3.5.5) (Leica Microsystems, Buccinasco, Italy) for Windows 10. All acquisition parameters were unchanged across the experimental group. Signal quantification was performed with ImageJ software v.154d (National Institutes of Health, Bethesda, MD, USA) for Windows 10 as ratio of mean fluorescence intensity to cell area, later normalized to the control level.

To evaluate mitochondrial morphology, MitoTracker™ Dyes for Mitochondria Labeling (M7512, Merck Life Science, Milan, Italy) was used according to producer instructions. Evaluation of mitochondrial parameters was assessed by using Mitochondrial Network Analysis tool (MiNA), which uses the FIJI distribution of the ImageJ platform, according to the previously described protocol [37]. Notably, the following parameters were analyzed: mitochondrial count, mitochondrial area, mean aspect ratio, mean form factor. Mitochondrial count represents the number of individual mitochondria within a cell, whereas mitochondrial area reflects the volume or total size of all mitochondria within a cell [38]. The morphological complexity of the mitochondrial network can be quantified through two descriptive parameters: the aspect ratio and the form factor. The aspect ratio, which provides an estimate of mitochondrial elongation, is defined as the ratio between the major and minor axes of an ellipse fitted to the organelle; a value of 1 corresponds to a perfectly circular profile. The form factor, calculated as [form factor = 1/4 × (area/perimeter2)], integrates information on both elongation and branching. Higher aspect ratio values are associated with mitochondria exhibiting more elaborate branching patterns [39]. The degree of colocalization between NPC1 and LAMP2, as well as between LC3 and Mitotracker, was assessed by calculating the Pearson’s correlation coefficient (PCC) using LAS X software (version 3.5.5, Leica Microsystems, Buccinasco, Italy). The antibody employed in this work for immunofluorescence are listed in Table 1.

### TUNEL Assay

SH-SY5Y were seeded on poly-L-Lysine coated-coverslips. After treatment, cells were fixed in order to detect and quantify apoptotic cells employing TUNEL assay (11684795910, Roche, Basilea, Switzerland), following the manufacturer’s instructions.

### Glutathione (GSH/GSSG) Quantification

Glutathione quantification assay was performed according to manufacturer instructions (MAK440, Merck Life Science, Milan, Italy).

### Statistical Analysis

Results are shown as mean ± standard deviation (SD). Normal distribution was assessed by using Shapiro–Wilk test, whereas the presence of potential significant outliers was evaluated by Grubb’s test. Unpaired Student’s t-test was used to compare mean values between two experimental groups. To evaluate three experimental groups, one-way analysis of variance (ANOVA) was performed along with Tukey’s *post hoc* test. *p* < 0.05 was considered as statistically significant. Statistical analysis was conducted with GraphPad Prism 8.4.2 (GraphPad, La Jolla, CA, USA) for Windows 10.

## Results

### p75NTR Modulation Mitigates PD Hallmarks in Rotenone-Treated SH-SY5Y Cells

**Fig. 1:**
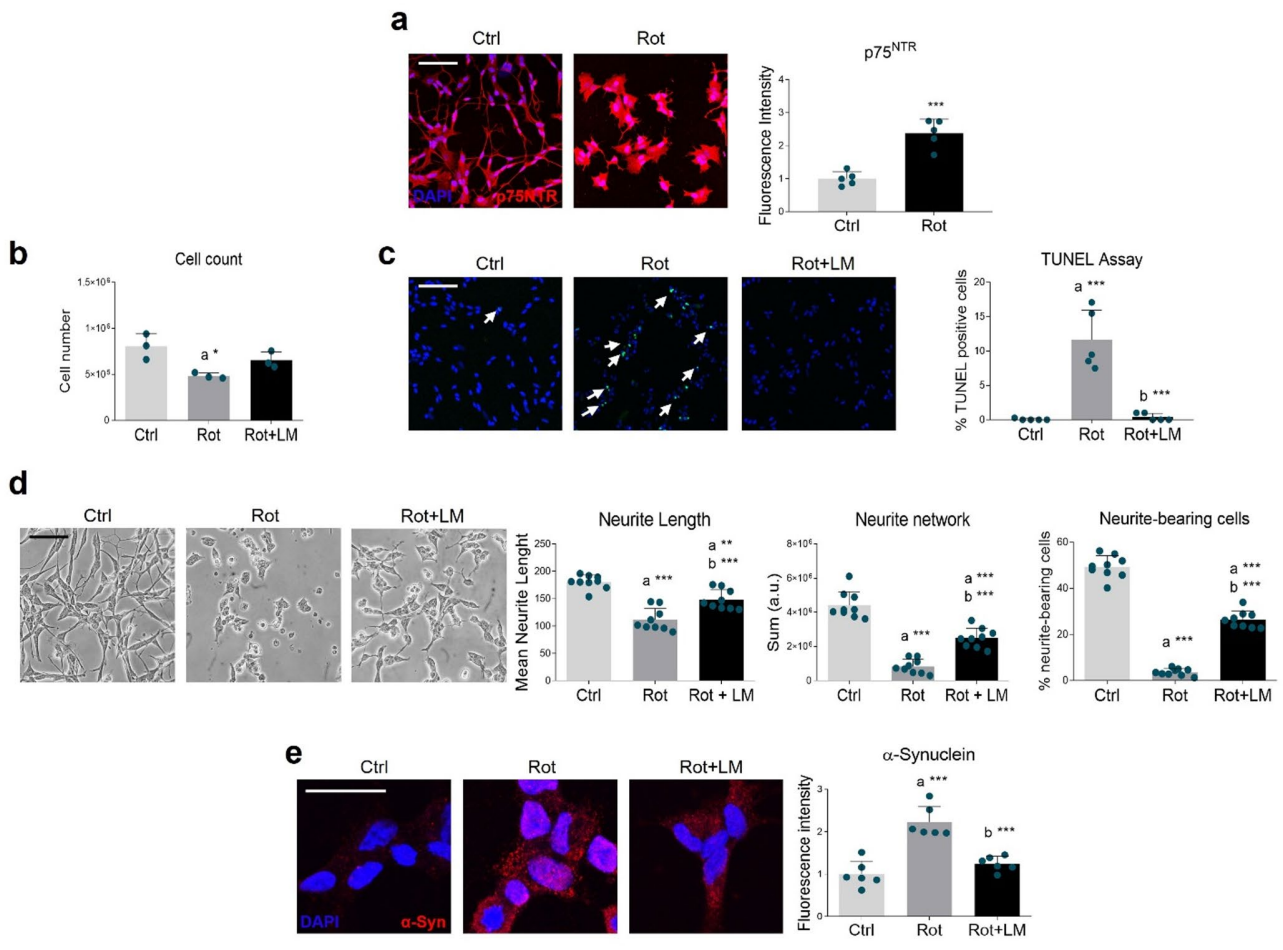
Effects of LM11A-31 on PD hallmarks. (**a**) Confocal microscopy representative images (left panel) and quantitative analysis (right panel) of p75NTR (red) on differentiated SH-SY5Y cells after treatments with vehicle (Ctrl, DMSO), 100 nM rotenone (Rot) for 24 h. DAPI (blue) was used to counterstain nuclei. *N* = 5. Data are represented as means ± SD. Statistical analysis was performed by using the Unpaired Student’s t-test. *** p < 0.001. Magnification: 40x. Scale bar = 50 μm. (**b**) Cell count, *N* = 3 and (**c**) TUNEL assay (green) of differentiated SH-SY5Y cell line treated with vehicle (Ctrl, DMSO), 100 nM rotenone (Rot) and co-treated with LM11A-31 (LM) at the dose of 500 nM for 24 h. DAPI (blue) was used to counterstain nuclei; *N* = 5. Magnification: 40x. Scale bar = 50 μm. (**d**) Representative brightfield images (left panel) and morphological analysis of the neurite length, neurite network and % of neurite-bearing cells (right panel) were conducted using ImageJ software (National Institutes of Health, Bethesda, MD, USA; Sun-Java). *N* = 9. (**e**) Confocal microscopy representative images of α-synuclein (red) on SH-SY5Y differentiated cell line treated as previously reported. DAPI (blue) was used to counterstain nuclei. *N* = 6. Magnification: 63x. Scale bar = 25 μm. Data are represented as means ± SD. The blue dots around the SD represent the different biological measurements. Statistical analysis was performed by using one-way ANOVA followed by Tukey’s *post hoc* test. “a” indicates statistical significance vs. Ctrl; “b” indicates statistical significance vs. Rot group. * *p* < 0.05, ** *p* < 0.01, *** *p* < 0.001

Abnormal p75NTR expression is reported in several neurological disorders, including PD [6, 7, 10]. Thus, we first examined whether p75NTR levels were altered in the PD cellular model employed in this study. Immunofluorescence analysis revealed that 24-hour exposure to rotenone significantly increased p75NTR expression (Fig. 1a). To investigate the consequences of p75NTR alterations in PD we used its synthetic modulator LM11A-31. We detected a significant reduction in cell number following rotenone treatment, which was attenuated by treatment with 100 nM LM11A-31 (Supplementary Fig. 1a). However, at this concentration, LM11A-31 failed to protect against the neuromorphological alterations caused by rotenone (Supplementary Fig. 1b). We reasoned that this absence of morphological protection might reflect an insufficient LM11A-31 dose to trigger maximal neuroprotective effects. To address this, experiments were repeated using 500 nM LM11A-31. The higher dose preserved the protective effect on cell number (Fig. 1b). This benefit appeared to be mediated through the modulation of apoptotic pathways, as confirmed by a significant reduction in the number of TUNEL-positive cells (Fig. 1c). The effects were not only restricted to cell viability, but also extended to morphological features: a dramatic neurite degeneration was observed upon rotenone stimulation, whereas p75NTR modulation partially prevented rotenone-dependent changes in neurite length, neurite network, and percentage of neurite-bearing cells (Fig. 1 d). Given that the most robust effects on both cell survival and neuronal morphology preservation were achieved with 500 nM LM11A-31, this concentration was selected for all subsequent experiments. α-Syn overexpression and aggregation are other key pathological hallmarks in PD, being the most abundant components of Lewy’s bodies [40]. Immunofluorescence analysis revealed that rotenone treatment elicited the build-up of α-Syn pucta-like staining, reminiscent of protein aggregation. Conversely, co-treatment with LM11A-31 markedly reduced such immunoreactivity pattern (Fig. 1e).

### p75NTR Modulation by LM11A-31 Counteracts Mitochondrial Alterations and Oxidative Stress

**Fig. 2:**
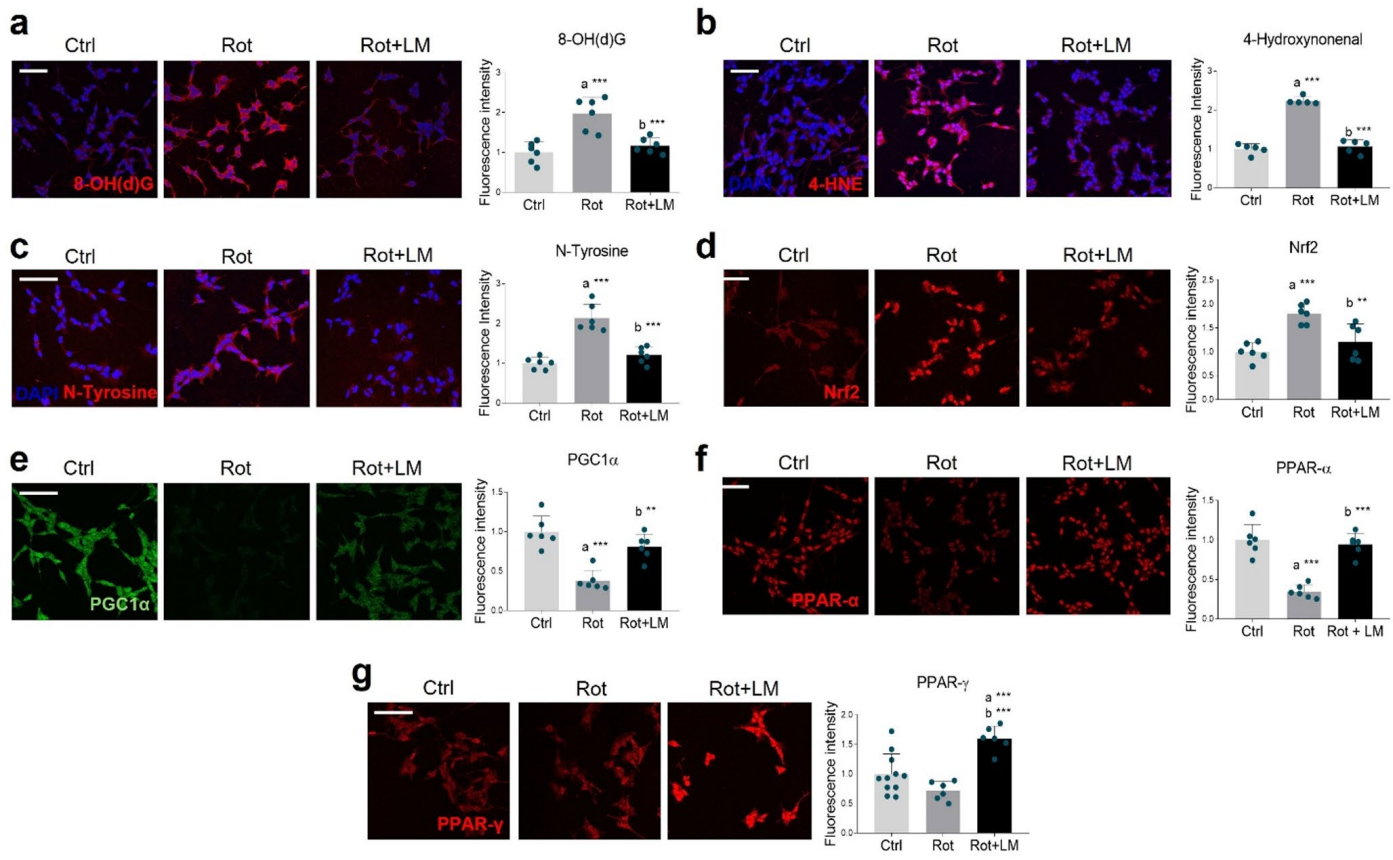
LM11A-31 effects on oxidative stress. Confocal microscopy representative images and fluorescent signal quantification of (**a**) 8-OH(d)G (red), (**b**) 4-HNE (red), (**c**) N-Tyrosine (red), (**d**) Nrf2 (red), (**e**) PGC-1α (green), (**f**) PPAR-α (red) and (**g**) PPAR-γ (red) in SH-SY5Y differentiated cell line treated with vehicle (Ctrl, DMSO), 100 nM rotenone (Rot) and rotenone with 500 nM LM11A-31 (Rot + LM) for 24 h. DAPI (blue) was used to counterstain nuclei. *N* = 5–11. The blue dots around the SD represent the different biological measurements. Statistical analysis was performed by using one-way ANOVA followed by Tukey’s *post hoc* test. “a” indicates statistical significance vs. Ctrl; “b” indicates statistical significance vs. Rot group. ** *p* < 0.01, *** *p* < 0.001. Magnification: 40x. Scale bar = 50 μm

Oxidative stress is one of the driving forces leading to dopaminergic neuronal death in PD. In this context, mitochondrial perturbances favor the production of ROS and reactive nitrogen species (RNS), which react with various biomolecules, causing damage to sub-cellular components [41]. We evaluated oxidative/nitrosative damage to nucleic acids, lipids and proteins by immunofluorescence, using antibodies against 8-hydroxy-(deoxy)-guanosine (8-OH(d)G)-, 4-hydroxynonenal (4-HNE)-, and nitrotyrosine (N-Tyrosine). As expected, rotenone treatment led to increased oxidative (Fig. 2a, b) and nitrosative (Fig. 2c) damage, which was normalized by pharmacological modulation of p75NTR.

Since ROS overproduction may be counteracted by antioxidant responses, we examined the expression of Nrf2, a master regulator of redox homeostasis. Immunofluorescence analysis revealed increased Nrf2 expression and nuclear localization upon rotenone treatment (Fig. 2d), suggesting the activation of a stress response. Co-treatment with the p75NTR modulator normalized Nrf2 immunoreactivity, consistent with the observation that LM11A-31 minimizes oxidative damage. Concerning other major players in antioxidant response and in mitochondrial biogenesis and function, peroxisome proliferator-activated receptor gamma coactivator 1-alpha (PGC-1α) (Fig. 2e) and peroxisome proliferator-activated receptor (PPAR)-α (Fig. 2f) were both markedly downregulated following rotenone treatment, while PPAR-γ expression appeared unchanged (Fig. 2g). However, LM11A-31 boosted the expression of all these transcription factors.

Mitochondrial dysfunction in dopaminergic neurons is a documented feature in PD and rotenone, acting as a potent and reversible inhibitor of mitochondrial complex I [13], is a well-known tool to model such alteration.

**Fig. 3:**
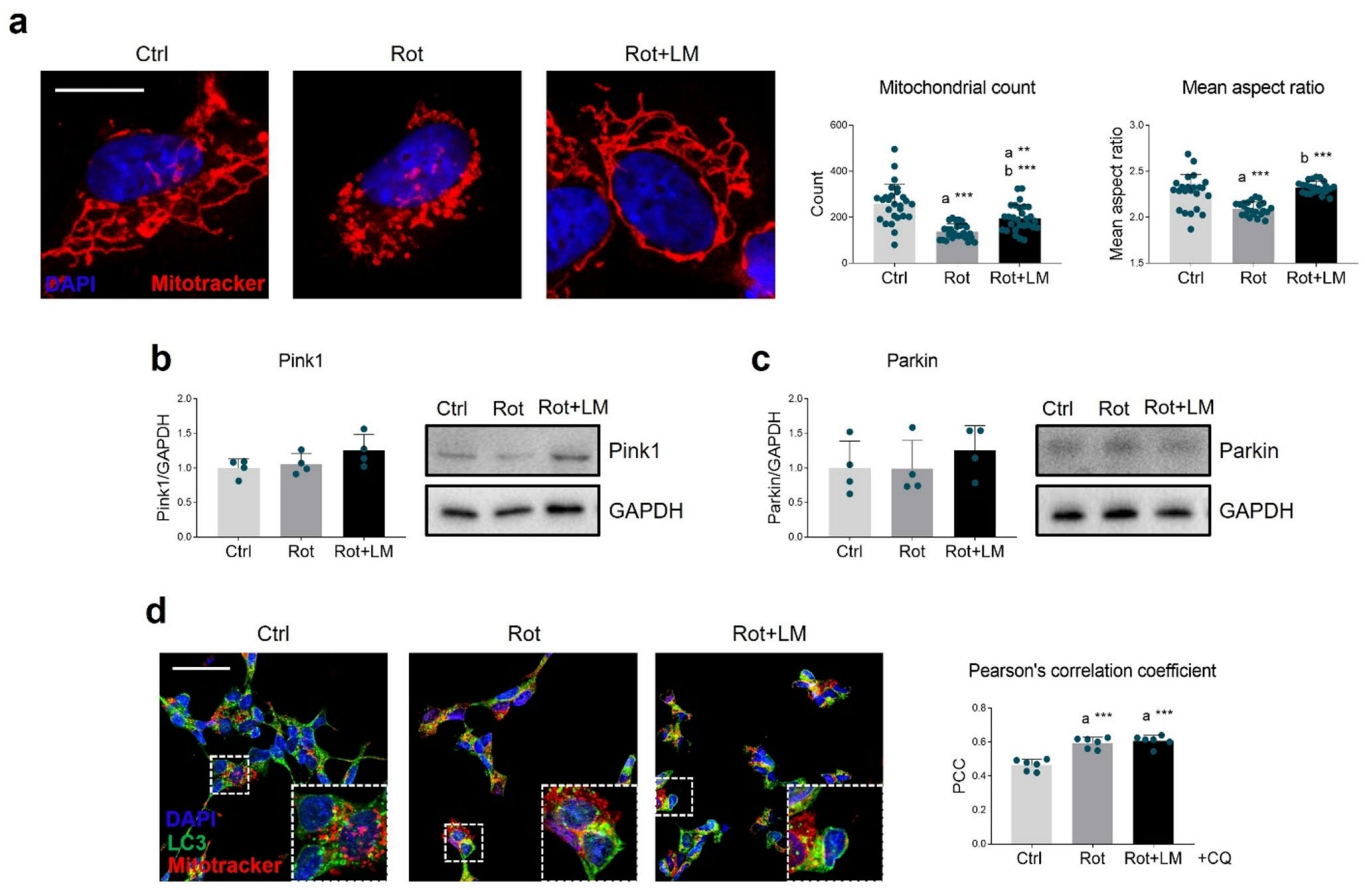
Effects of LM11A-31 on mitochondrial homeostasis. (**a**) Representative images of Mitotracker (red) and quantification of morphological indicators (mitochondrial count, mean aspect ratio) in SH-SY5Y cells treated with vehicle (Ctrl, DMSO), 100 nM rotenone (Rot) and rotenone with 500 nM LM11A-31 (Rot + LM) for 24 h. DAPI (blue) was used to counterstain nuclei. *N* = 23–27 images were analyzed from 6 independent experiments. Magnification: 100x. Scale bar = 10 μm. (**b**,** c**) Representative Western blot and densitometric analysis of Pink1 and Parkin proteins in SH-SY5Y cells treated as above. GAPDH was used as internal loading control. *N* = 4. (**d**) Immunofluorescence (left panel) showing co-immunofluorescence between Mitotracker (red) and LC3 (green) in SH-SY5Y treated as in (**a**). Chloroquine (20 µM) was added 4 h prior cell fixation to inhibit autophagosome fusion with lysosomes, thereby enhancing the visualization of mitophagy. Pearson’s correlation coefficient (PCC, right panel) was calculated to estimate protein colocalization. *N* = 6. Magnification: 40x. Scale bar = 50 μm. Data are represented as means ± SD. The blue dots around the SD represent the different biological measurements. Statistical analysis was performed by using one-way ANOVA followed by Tukey’s post hoc test. *** *p* < 0.001. “a” indicates statistical significance vs. Ctrl

In our model, rotenone-treated SH-SY5Y cells exhibited a marked reduction in mitochondrial abundance and pronounced fragmentation. While control cells displayed elongated, tubular, and branched mitochondria, rotenone exposure led to the predominance of donut/blob-shaped structures. These morphological alterations were quantitatively confirmed using the MiNA tool: mitochondrial count, mean aspect ratio, and mean area were all significantly reduced upon rotenone treatment (Figs. 3a, Supplementary Fig. 2a). In contrast, LM11A-31 prevented mitochondrial abnormalities by restoring mitochondrial number and area, as well as increasing both mean aspect ratio and mean form factor (Figs. 3a, Supplementary Fig. 2a, b).

Considering the observed changes in mitochondrial morphology, we hypothesized the involvement of mitophagic turnover as a possible resilience mechanism. To explore this, we evaluated the expression of PTEN-induced kinase 1 (PINK1) and Parkin, which are two especially relevant mitophagy regulators in PD. Western blot analysis did not reveal any changes in PINK1 (Fig. 3b) and Parkin (Fig. 3c) protein expression across the experimental groups. However, combined Mitotracker staining and immunofluorescence for the autophagosomal marker microtubule-associated protein 1 A/1B-light chain 3 (LC3) revealed increased colocalization of the two signals in rotenone-treated SH-SY5Y (Fig. 3d), suggesting enhanced mitophagic activity. LM11A-31 treatment did not significantly modulate this process.

### LM11A-31 Treatment Modulates Both Antioxidant and Pro-Oxidant Pathways

**Fig. 4:**
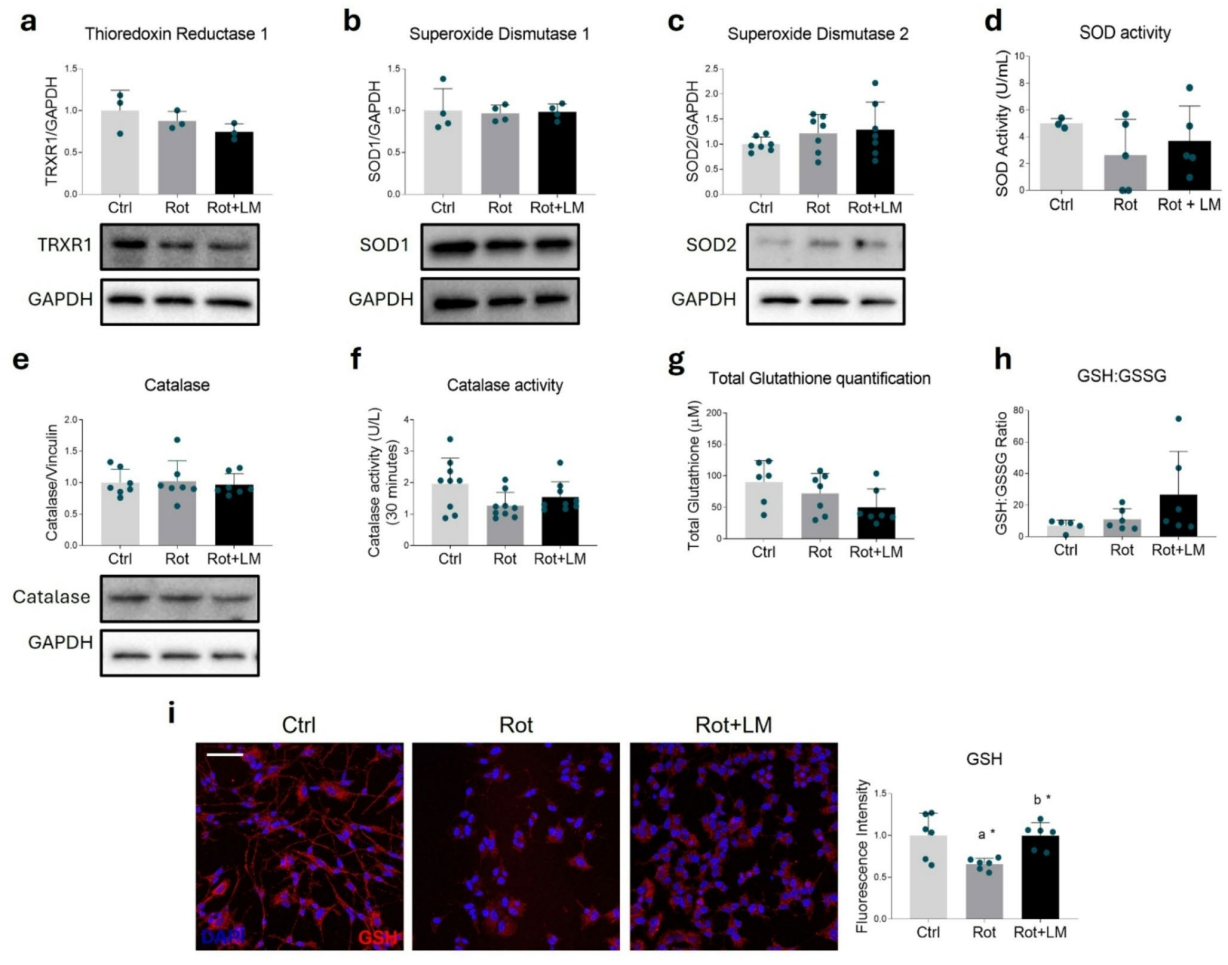
LM11A-31 effects on antioxidant scavengers. Representative Western blot and densitometric analysis of TrxR1 (**a**), SOD1 (**b**), SOD 2 (**c**), differentiated SH-SY5Y treated with DMSO (Ctrl), 100 nM rotenone (Rot) or rotenone with 500 nM LM11A-31 (Rot + LM) for 24 h. GAPDH was chosen as loading control. *N* = 3–7. (**d**) Total SOD activity was measured in U/mL. *N* = 3–5 biological replicates. (**e**) Representative Western blot and densitometric analysis of Catalase; GAPDH was chosen as loading control. *N* = 7. (**f**) Catalase activity was measured in U/L of conversion in 30 min. *N* = 9. (**g**) Total amount of GSH, expressed in µM concentration. *N* = 6–7 biological replicates. (**h**) Ratio between reduced (GSH) and oxidized (GSSG) glutathione. *N* = 5–6 different experiments. (**i**) Confocal microscopy representative images and fluorescent signal quantification of Glutathione (red) in SH-SY5Y cells treated as mentioned above. DAPI (blue) was used to counterstain nuclei. *N* = 6. Magnification: 40x. Scale bar = 50 μm. Data are represented as means ± SD. The blue dots around the SD represent the different biological measurements. Statistical analysis was performed by using one-way ANOVA followed by Tukey’s post hoc test. “a” indicates statistical significance vs. Ctrl; “b” indicates statistical significance vs. Rot group. * *p* < 0.05

Nrf2 and PPARs elicit cell response to oxidative stress by upregulating many enzymes involved in ROS scavenging, whose expression was evaluated in this work. Thioredoxin reductase 1 (TrxR1) levels remained unchanged among the experimental groups (Fig. 4a). Similarly, no significant variations were observed in the expression of Superoxide Dismutase (SOD) 1 and 2 following rotenone exposure or LM11A-31 treatment (Fig. 4b, c), nor in total SOD enzymatic activity (Fig. 4d). Catalase expression was also unaffected by the treatments (Fig. 4e), and no significant changes in its activity were observed across groups, although a slight downward trend was noted in rotenone-treated cells (Fig. 4f).

Regarding non-enzymatic ROS scavengers, total glutathione (GSH) levels were unchanged across all experimental conditions (Fig. 4g), as well as the ratio of reduced to oxidized glutathione (GSH: GSSG) (Fig. 4h). Concurrently, no variations were reported for GSH biosynthetic enzymes (γ-glutamate-cysteine ligase and GSH synthase) nor in GSH-utilizing enzymes such as GSH peroxidase (Gpx)1 and GSH reductase (GSR) (Supplementary Fig. 3a–d). GSH also exerts protective effects in pro-oxidant environments by binding proteins through post-translational modifications, specifically through S-glutathionylation. Immunofluorescence analysis for GSH-bound proteins revealed a significant reduction in immunoreactivity upon rotenone treatment. Notably, LM11A-31 effectively restored protein S-glutathionylation (Fig. 4i).

**Fig. 5:**
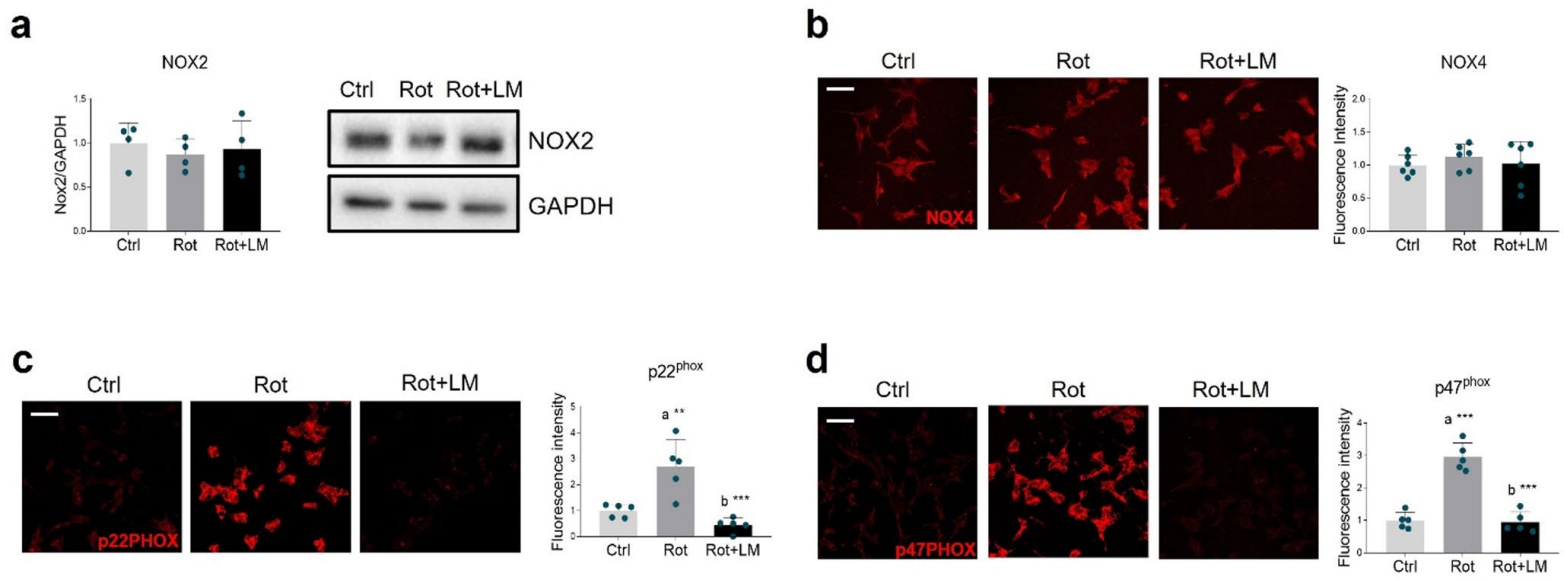
Effects of p75NTR modulation on NADPH oxidase complex in rotenone-treated SH-SY5Y cells. (**a**) Representative Western blot and densitometric analysis of NOX2 subunit in differentiated SH-SY5Y treated with DMSO (Ctrl), 100 nM rotenone (Rot) or rotenone with 500 nM LM11A-31 (Rot + LM) for 24 h. GAPDH was chosen as loading control. *N* = 4 biological replicates. (**b–d**) Representative immunofluorescence images and respective signal quantification of (**b**) NOX4 (red), (**c**) p22^PHOX^ (red) and (**d**) p47^PHOX^ (red) NADPH oxidase subunits in SH-SY5Y neuronal cells treated as in (**a**). *N* = 5–6. Magnification: 40x. Scale bar = 50 μm. Data are expressed as mean ± SD. The blue dots around the SD represent the different biological measurements. Statistical analysis was assessed using the one-way ANOVA test, followed by Tukey’s *post hoc* test. Statistical significance is indicated as follows: ** *p* < 0.01; *** *p* < 0.001. “a” indicates statistical significance vs. Ctrl group; “b” indicates statistical significance vs. Rot group

In addition to mitochondria, cells rely on other endogenous sources of ROS production, among which NADPH oxidase (NOX) is the most extensively studied in PD pathogenesis [42]. While no variations were observed in the expression of NOX2 (Fig. 5a) and NOX4 catalytic subunits (Fig. 5b), rotenone exposure increased the immunopositivity associated with p22^PHOX^ (Fig. 5c) and p47^PHOX^ (Fig. 5d) regulatory subunits. Interestingly, LM11A-31 significantly downregulated the expression of both subunits (Fig. 5c, d).

### p75NTR Modulation Reverses Rotenone-Associated Cholesterol Dysmetabolism

**Fig. 6:**
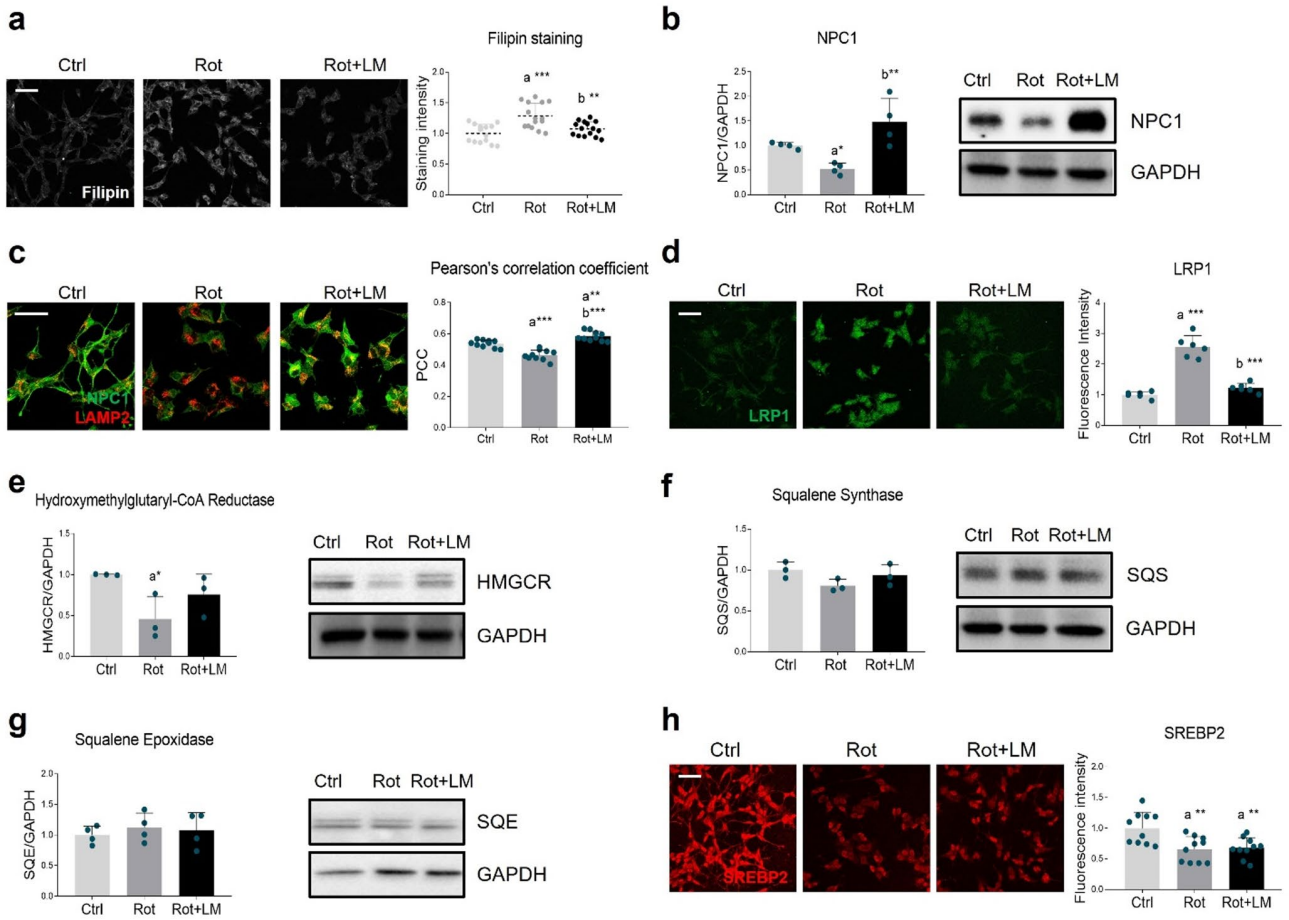
Impact of LM11A-31 on cholesterol metabolism in rotenone-treated cells. (**a**) Representative images (left panel) and quantification of filipin staining (right panel) performed on differentiated SH-SY5Y cells after treatments with vehicle (Ctrl, DMSO), 100 nM rotenone (Rot) and rotenone with 500 nM LM11A-31 (Rot + LM) for 24 h. *N* = 15. Magnification: 40x. Scale bar = 50 μm. (**b**) SH-SY5Y treated as in A were subjected to Western blot and densitometric analysis for NPC1. GAPDH was used as housekeeping protein for loading control. *N* = 4 biological replicates. (**c**) Co-immunofluorescence analysis performed on SH-SY5Y neuronal cells treated as previously reported, by using antibodies against NPC1 (green) and LAMP2 (red). Pearson’s correlation coefficient (PCC, right panel) was calculated to estimate the protein colocalization. *N* = 10. Magnification: 40x. Scale bar = 50 μm. (**d**) Representative immunofluorescence images of LRP1 protein (green) in differentiated neuronal cells treated as previously described. *N* = 6. Magnification: 40x. Scale bar = 50 μm. (**e**-**g**) Representative immunoblot and densitometric analysis of HMGCR, SQS and SQE. GAPDH was used as internal loading control. *N* = 3–4 independent experiments. (**h**) Immunofluorescence for SREBP2 (red) performed of SH-SY5Y cells. *N* = 10 biological replicates. Magnification: 40x. Scale bar = 50 μm. Data are represented as means ± SD. The blue dots around the SD represent the different biological measurements. Statistical analysis was performed by using one-way ANOVA followed by Tukey’s post hoc test. * *p* < 0.05, ** *p* < 0.01, *** *p* < 0.001. “a” indicates statistical significance vs. Ctrl; “b” indicates statistical significance vs. Rot group

Recent findings demonstrated that rotenone exposure disrupts cholesterol metabolism by downregulating the expression of NPC1, thereby promoting cholesterol build-up into lysosomes [15]. Consistently, we observed free cholesterol accumulation in rotenone-treated cells, which was attenuated by LM11A-31 stimulation (Fig. 6a). To investigate whether the effects were dependent on intracellular cholesterol trafficking, we performed Western blot and immunofluorescence analysis, showing that NPC1 expression (Fig. 6b) and localization to lysosomes (Fig. 6c) were significantly reduced upon rotenone exposure. Such alterations were prevented by p75NTR modulation via LM11A-31 (Fig. 6b, c). Cholesterol accumulation in rotenone-treated cells may also be related to its enhanced uptake by low-density lipoprotein receptor-related protein 1 (LRP1), which indeed significantly increased in response to rotenone, while being normalized by LM11A-31 (Fig. 6d). To further elucidate the molecular mechanisms controlling cholesterol metabolism, we examined the expression of the main enzymes involved in the biosynthetic pathway. The levels of the key and rate-limiting enzyme 3-hydroxy-3-methylglutaryl-CoA reductase (HMGCR) were reduced in rotenone-treated cells but restored following p75NTR modulation (Fig. 6e). Differently, no significant changes were observed in the expression of squalene synthase (SQS) (Fig. 6f) and squalene epoxidase (SQE) (Fig. 6g). Finally, immunofluorescence analysis of sterol regulatory element-binding protein 2 (SREBP-2), a master regulator of cholesterogenic expression, showed a significant downregulation in response to rotenone that was not reversed by LM11A-31 (Fig. 6h).

## Discussion

During last decade, p75NTR has emerged as a promising therapeutic target in several neurodegenerative conditions, including AD, HD and ALS [43]. However, information about its modulation in PD remain elusive. Here, we bring evidence that pharmacological modulation of p75NTR by LM11A-31 prevents parkinsonian features in rotenone-treated SH-SY5Y cells. Rotenone was applied at a concentration of 100 nM, a dose widely used in SH-SY5Y and SK-N-SH cells to induce PD-like molecular features, including mitochondrial dysfunction, oxidative stress, and apoptosis [44–46]. Immunofluorescence analysis showed p75NTR upregulation upon rotenone exposure, corroborating previous findings on receptor alterations in PD [9, 18]. Treatment with 500 nM LM11A-31 enhanced cell viability, reduced apoptosis, restored neuromorphological aberrations, and lowered α-syn levels, when compared to rotenone-treated cells. In line with previous reports [47, 48], rotenone exposure led to mitochondrial fragmentation, which was reversed by LM11A-31. Despite unaltered PINK1 and Parkin levels, mitophagy was induced in rotenone-treated cells. This may represent a compensatory mechanism aimed at removing damaged organelles which, however, was not influenced by LM11A-31. The regulation of mitochondrial dynamics can be inferred from quantitative assessments of morphological parameters. Reduction in mitochondrial mean area, aspect ratio and form factor are indicative of increased fission and enhanced fragmentation, consistent with the activation of mitophagy to eliminate dysfunctional mitochondria [39, 49]. Mitophagy, while essential for organelle turnover, functions in opposition yet in complementarity with mitochondrial biogenesis, the latter being responsible for the growth and elongation of pre-existing mitochondria to maintain or restore mitochondrial biomass [49]. Our findings point to an imbalance between mitophagy and biogenesis in rotenone-treated cells. Specifically, the reduction in mean aspect ratio suggests enhanced organelle fragmentation and mitophagy, while the concomitant decrease in the mean area implies that mitophagy prevails over mitochondrial biogenesis. Consistently, the expression of key transcriptional regulators of mitochondrial biogenesis, namely PPAR-α and PGC-1α, was significantly downregulated following rotenone exposure. In contrast, LM11A-31 treatment increased mitochondrial counts, area, aspect ratio, and form factor, indicating that the compound re-establishes the balance between mitophagy and biogenesis by promoting mitochondrial renewal.

Rotenone exposure led to increased expression of oxidative stress markers like 8-OHdG, 4-HNE, and N-tyr, in line with its well-known action as a mitochondrial complex I inhibitor and inducer of ROS overproduction. Notably, reduction of S-glutathionylation reported in our study may further exacerbate protein vulnerability to oxidative damage [50], contributing to the observed cellular stress. Although not statistically significant, we also observed a downward trend in catalase activity following rotenone treatment, which could be consistent with previous findings in SH-SY5Y cells exposed to similar concentrations [44]. Differences in culture conditions (i.e. FBS concentration, differentiated vs. undifferentiated cells), could have influenced both the biological magnitude of the effect and the statistical power of the analysis.

Additionally, our data on rotenone-dependent increased expression of NOX regulatory subunits p22PHOX and p47PHOX suggest that redox imbalance in this model may also result from upregulation of pro-oxidant systems. This observation aligns with the concept that mitochondrial ROS production can further stimulate NOX activation, thus establishing a vicious cycle of oxidative stress [51]. Indeed, ROS-sensitive transcription factors have been reported to regulate the expression of genes encoding NOX subunits, further amplifying this pro-oxidant feedback loop [52]. In the specific context of PD, Keeny and colleagues corroborate our findings, highlighting the involvement of NOX2 complex in the early stages of PD, where its activation contributes to oxidative stress-dependent post-translational modification of α-syn, ultimately resulting in its aggregation [53]. In line with this, other studies have demonstrated that rotenone-induced neurotoxicity in SH-SY5Y cells involves NOX activation [54].

Importantly, LM11A-31 treatment significantly reduced oxidative damage. This effect was associated with restored protein S-glutathionylation and decreased expression of the NOX regulatory subunits p22PHOX and p47PHOX. The improvement in redox homeostasis induced by LM11A-31 also correlates with changes in intracellular distribution of Nrf2, a key transcription factor regulating antioxidant responses. Indeed, upon oxidative stress, Nrf2 becomes stabilized and accumulates in the nucleus, whereas under controlled redox conditions, it undergoes Keap1-mediated proteasomal degradation [55].

Overall, the protective effects of LM11A-31 on mitochondrial integrity and oxidative stress may, at least in part, result from the upregulation of transcription factors involved in redox regulation and mitochondrial biogenesis, such as PPAR-α, PPAR-γ, and their co-activator PGC-1α. These pathways may collectively contribute to the observed improvements in mitochondrial morphology, as well as overall cellular resilience to oxidative damage.

Cholesterol is a critical component of biological membranes contributing to their structural integrity and stability, while supporting synapse formation and neurotransmitter release. In line with previous evidence [15], rotenone-treated cells displayed intracellular cholesterol overload, accompanied by reduced NPC1 expression and impaired lysosomal localization, indicative of disrupted cholesterol trafficking in the PD-like phenotype. Additionally, HMGCR expression was markedly decreased, suggesting that impaired sterol biosynthesis may be a compensatory feedback response to the intracellular cholesterol buildup. Remarkably, LM11A-31 counteracted these alterations, by restoring HMGCR expression, NPC1 levels and correct lysosomal distribution. In addition to its antioxidant properties, PPAR-α regulates cholesterol metabolism by activating the transcription of several key genes, including those encoding HMGCR and NPC1. Consistently, PPAR-α ligands have been shown to enhance the expression of both proteins [56–58]. As rotenone exposure downregulated PPAR-α expression and LM11A-31 treatment restored it, future investigation will be necessary to determine whether the upregulation of this transcription factor directly contributes to the recovery of cholesterol homeostasis. Although further studies are required to dissect the molecular mechanisms, it can be speculated that p75NTR modulation may rescue cholesterol redistribution in the cell, thus ameliorating lysosomal availability for degrading protein aggregates and damaged organelles. Additionally, recent findings have also indicated that hypercholesterolemia, as well as cholesterol accumulation into lysosomes, alters physical properties of mitochondrial membranes, contributing to ROS leakage and the subsequent spread of oxidative stress [59, 60]. Collectively, these lines of evidence suggested that the cytoprotective effects of LM11A-31 may depend on the interplay between cholesterol homeostasis, redox balance and mitochondrial turnover.

It is worth noting that LM11A-31 successfully entered phase IIa of a clinical trial for AD, showing adequate safety profile and bioavailability [29]. Furthermore, LM11A-31 has been demonstrated to restore Akt expression in the *striatum* while inhibiting c-Jun N-terminal kinase (JNK) pathways in R6/2 animal models, resulting in increased dendritic spine density and reduced inflammation [61]. These notions, along with the results shown in this work, suggest that p75NTR pharmacological modulation might be a valuable therapeutic approach not only in counteracting AD but also in other neurodegenerative conditions, such as PD and HD.

Further studies are required to expand the characterization of LM11A-31 effects in additional PD models. Although there are no single experimental models that can fully replicate the complexity of human PD, rotenone exposure has emerged as one of the most reliable environmental toxins for mimicking sporadic PD-like features. Notably, unlike 6-OHDA, MPTP, or Paraquat, rotenone better recapitulates key aspects of PD physiopathology, including the aggregation of endogenous wild-type α-synuclein and the formation of Lewy bodies. Furthermore, rotenone induces dopaminergic neuronal loss in the SNpc more prominently than in other brain regions [62, 63]. Despite the advantages offered by the rotenone-induced model, PD is widely recognized as a multifactorial disease, with both genetic and environmental factors contributing to its onset [64]. Therefore, investigating LM11A-31 in more sophisticated experimental models, such as those combining rotenone exposure with PD-related genetic mutations [65, 66], may provide deeper insights into its therapeutic potential.

Additional studies are also needed to elucidate the molecular mechanisms underlying LM11A-31 action, particularly regarding its regulation of oxidative stress and cholesterol metabolism. While current evidence supports the involvement of p75NTR modulation in both redox balance [19] and intracellular cholesterol homeostasis [1], the precise signaling pathways remain to be fully defined and may vary depending on the cell type and the particular pathophysiological context. Given the central role of mitochondrial dysfunction in PD pathogenesis, special attention should also be devoted to the influence of LM11A-31 on mitochondrial dynamics, including fission and fusion processes. Furthermore, while cell culture systems may offer valuable insights by modeling a simplified version of the pathology, future research should aim to validate these findings in more complex biological models, such as in vivo models, to fully evaluate the therapeutic efficacy and safety profile of LM11A-31.

## Supplementary Information

Below is the link to the electronic supplementary material.

Supplementary Material 1

## Data Availability

No datasets were generated or analyzed during the current study. The data that support the findings of this study are available from the corresponding author upon reasonable request.
