# Supplementary material for: p75NTR Modulation by LM11A-31 Counteracts Oxidative Stress and Cholesterol Dysmetabolism in a Rotenone-Induced Cell Model of Parkinson’s Disease
Source: Neurochem Res. 2025 Oct 4;50(5):315. doi: 10.1007/s11064-025-04569-7 (PMC12496268; doi:10.1007/s11064-025-04569-7)
Supplement: Supplementary file 1 — Supplementary Material 1 [file 11064_2025_4569_MOESM1_ESM.pdf]

Neurochemical Research

p75NTR modulation by LM11A-31 counteracts oxidative stress and cholesterol dysmetabolism in a rotenone-induced cell model of Parkinson's Disease

Daniele Pensabene[1], Noemi Martella[1], Giuseppe Scavo[1], Emanuele Bisesto[1], Francesca Cavicchia[1], Mayra Colardo[1], Michela Varone[1], Sandra Moreno[2,3], Marco Segatto[1*]

[1] Department of Biosciences and Territory, University of Molise, Contrada Fonte Lappone, 86090, Pesche, Italy;
[2] Department of Science, University Roma Tre, Viale Guglielmo Marconi 446, 00146, Rome, Italy;
[3] Lab of Neurodevelopmental Biology, Neurogenetics and Molecular Neurobiology, IRCCS Fondazione Santa Lucia, Via del Fosso di Fiorano 64, 00143, Rome, Italy

*Correspondence: marco.segatto@unimol.it

Supplementary information

Supplementary Figure 1

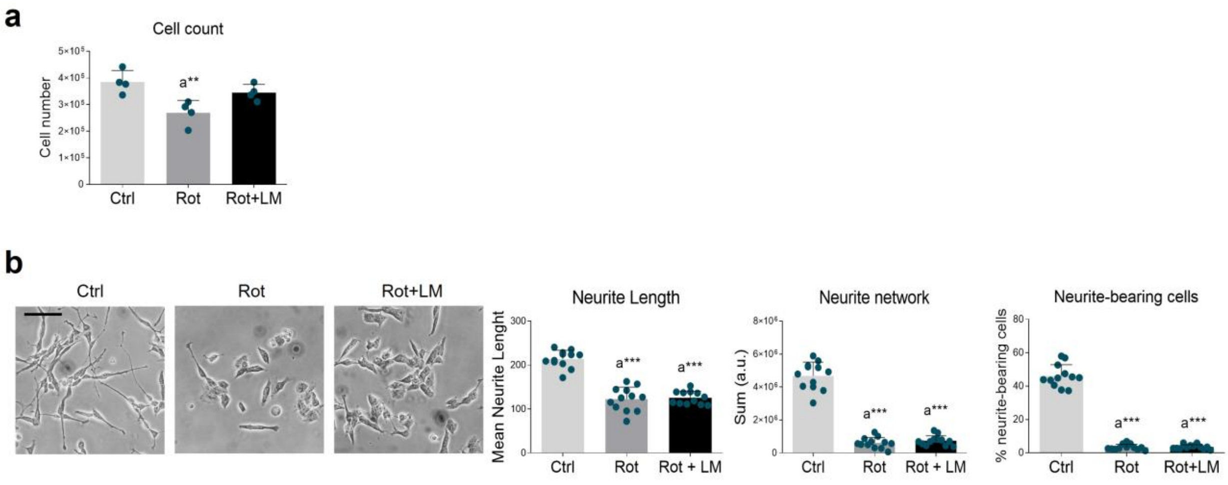

Supplementary Figure 1 (**a**) Cell count on differentiated SH-SY5Y cells treated with vehicle (Ctrl, DMSO), 100 nM rotenone (Rot) or rotenone with 100 nM LM11A-31 (Rot+LM) for 24 h. N=4 independent experiments. (**b**) Representative brightfield images (left panel) and morphological analysis of the neurite length, neurite network and % of neurite-bearing cells (right panel) were conducted on SH-SY5Y differentiated cells treated as reported in (a). N=9 biological replicates. Data are represented as means ± SD. The blue dots around the SD represent the different biological measurements. Statistical analysis was performed by using one-way ANOVA followed by Tukey's post hoc test. "a" indicates statistical significance vs Ctrl, "b" indicates statistical significance vs Rot. * $p < 0.05$, ** $p < 0.01$.

Supplementary Figure 2

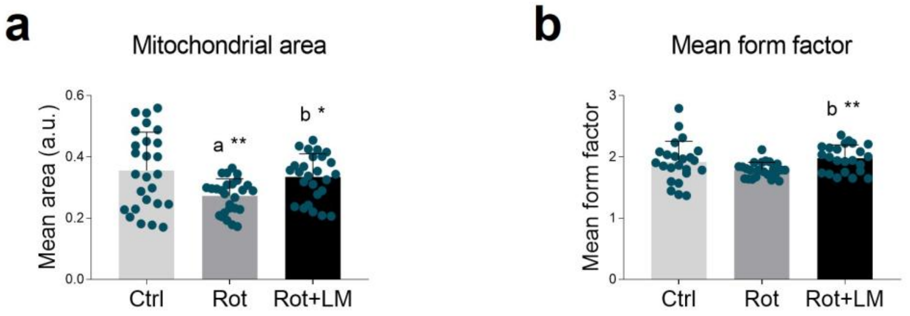

Supplementary Figure 2 (**a**) Statistical analysis of mitochondrial area and (**b**) mean form factor on differentiated SH-SY5Y cells treated with vehicle (Ctrl, DMSO), 100 nM rotenone (Rot) or rotenone with 500 nM LM11A-31 (Rot+LM) for 24 h. N=23-26 images were analyzed from 6 independent experiments. Data are represented as means ± SD. Statistical analysis was performed by using one-way ANOVA followed by Tukey's post hoc test. "a" indicates statistical significance vs Ctrl. *** $p < 0.001$.

Supplementary Figure 3

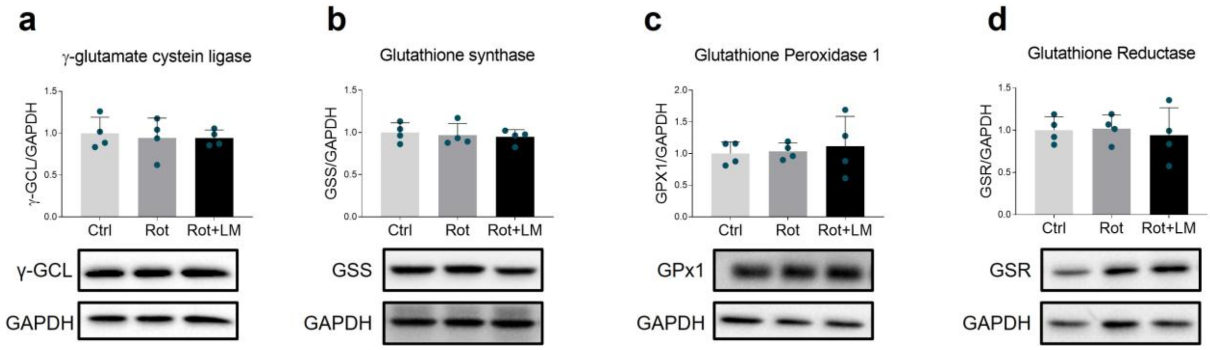

Supplementary Figure 3 Representative Western blots and densitometric analysis of (**a**) γ-GCL, (**b**) GSS, (**c**) GPX1, (**d**) GSR in differentiated SH-SY5Y treated with DMSO (Ctrl), 100 nM rotenone (Rot) or rotenone with 500 nM LM11A-31 (Rot+LM) for 24h. GAPDH was chosen as loading control. N=4 biological replicates. Data are expressed as mean ± SD. Statistical analysis was assessed using the one-way ANOVA test, followed by Tukey's *post hoc* test.
